# Supplementary material for: Superconducting phase diagram of H3S under high magnetic fields
Source: Nat Commun. 2019 Jun 7;10:2522. doi: 10.1038/s41467-019-10552-y (PMC6555813; doi:10.1038/s41467-019-10552-y)
Supplement: Supplementary file 1 — Supplementary Information [file 41467_2019_10552_MOESM1_ESM.pdf]

Supplementary Information

for

Superconducting Phase-Diagram of H_3S under High Magnetic Fields

S. Mozaffari et al.

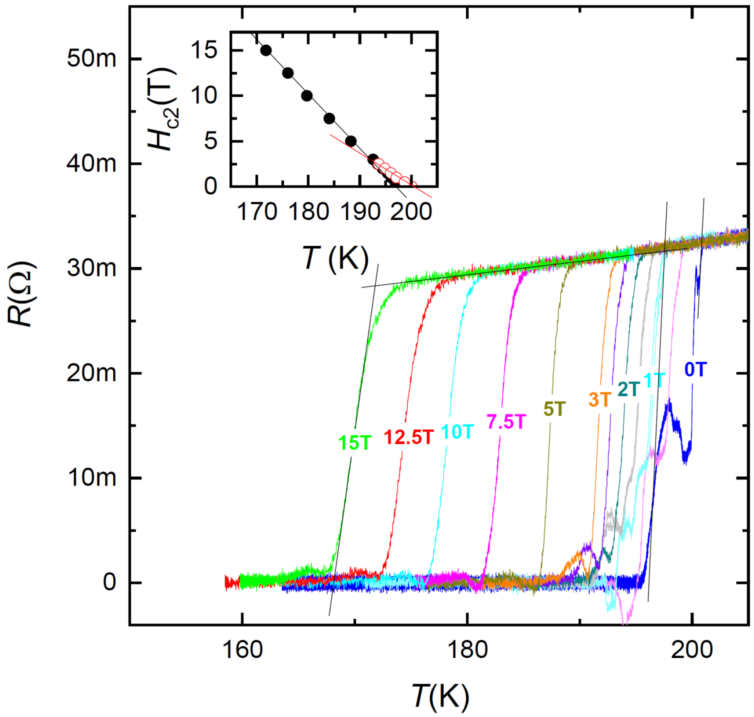

Supplementary Figure 1. Resistance as a function of the temperature for the sample under 155 GPa and applied magnetic fields up to 15 T. The zero field resistance trace displays the first onset of superconductivity at 201 K, followed by multiple additional transitions, signalling the presence of several superconducting phases with slightly different T_c's. Here, H_{c2} is defined as the intersection between an extrapolation of the resistance in the normal state and a line having the slope of the resistive transition at its middle point, as illustrated by black lines. We plotted the temperature dependence of the leading edge of the H_{c2} transition (red open circles in the inset), which extrapolates to 201 K, and the trailing edge of the transition (black filled circles), which extrapolates to 197 K. The leading edge transition temperature decreases with increasing magnetic field faster than the trailing edge transition temperature, until the leading edge merges into the trailing one above a modest field of just $\mu_0 H = 3$ T. Similar behavior was also observed in LaH_{10}[1]. This behaviour likely results from inhomogeneities in the superconducting sample, where the superconductivity first sets across weak inter-grain links and initially easily suppressed by magnetic field. We choose T_c=197 K for the WHH fit, as it represents the phase that dominates the high field H_{c2} behaviour of the sample under 155 GPa.

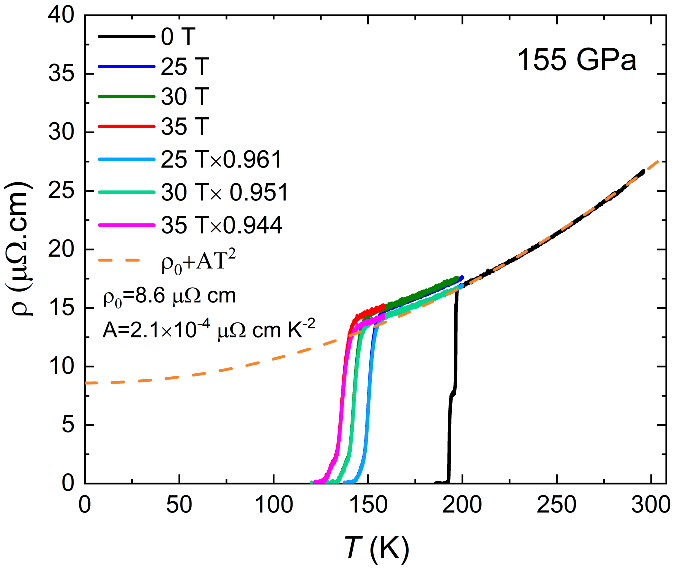

Supplementary Figure 2. Resistivity of the 155 GPa sample as a function of temperature at several constant field values between 0 T and 35 T. The sample's sheet resistance was determined using the Van der Pauw method. The resistivity of the 155 GPa sample was then determined from its sheet resistance and its thickness, estimated as $t = (2 \pm 1)\,\mu\mathrm{m}$. To compensate for the magnetoresistance and extend the usable temperature range of the fit, the resistivity under field was re-scaled to obtain the best overlap with $\mu_0 H = 0$ T trace. This scaling does not account for the temperature dependence of the magnetoresistance, but the latter is rather small, as indicated by the good overlap of the 25 T and 30 T scaled traces. At some point in time, after this data set was collected, the DAC experienced a drop in $\rho(T)$ with a concomitant increase in T_{c}, as shown in the main text. We find that $\rho(T)$ exhibits supralinear behavior well described by a $\rho \propto T^2$ leading term with pre-factor $A = 2.1 \times 10^{-4}\,\mu\Omega\,\mathrm{cm}\,\mathrm{K}^{-2}$. A quadratic temperature dependence for the resistivity is usually ascribed to inelastic electron-electron scattering within the Fermi liquid (FL) model. Charge transport in most metals is dominated by electron-phonon scattering at such high temperatures, while the FL T^2 term is observed at much lower temperatures where the phonons are depopulated. A rough estimate of the temperature-dependent electron-electron relaxation rate in FL model yields $\tau^{-1} \simeq (k_{\mathrm{B}}T)^2/(\hbar E_{\mathrm{F}}) = 10^{11}$ s^{-1}, where k_B is the Boltzmann constant and E_F is the Fermi energy in a metal $\sim$ 5 eV. Using $\rho = m/(e^2 n\tau)$, and taking resistivity value of $30\,\mu\Omega\,\mathrm{cm}$ and the Hall carrier density estimated as $8 \times 10^{28}\,\mathrm{m}^{-3}$ (assuming a single band model), the observed scattering rate is $\tau^{-1} \sim 10^{14}$ s^{-1}, 3 orders of magnitude higher than FL estimate, indicating that T^2 term in $\rho(T)$ is likely due to coupling to high-energy phonon modes[2, 3].

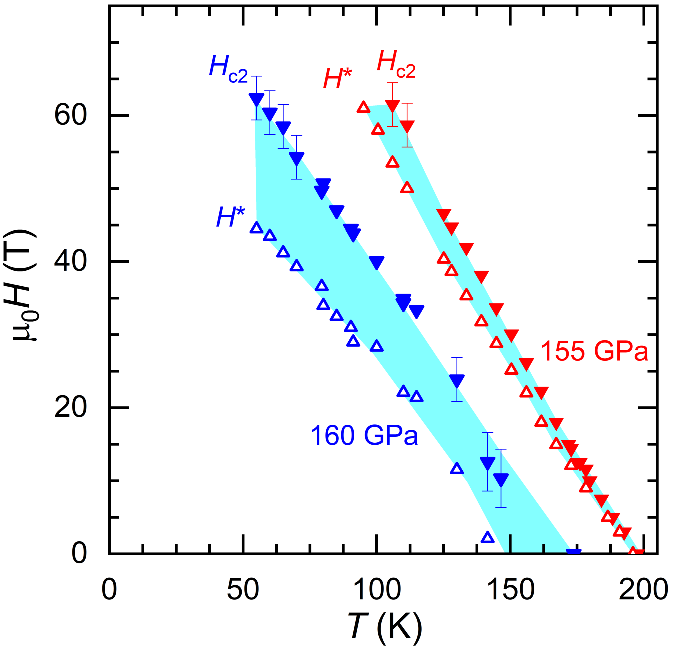

Supplementary Figure 3. The values of H_{c2} (filled triangles) and of $H^*(T)$ (open triangles) are plotted as a function of the temperature T for the samples under 155 GPa (red) and 160 GPa (blue). Error bars are standard deviation in linear extrapolation of the resistance in the normal state and the slope of the resistive transition used in H_{c2} determination. While H_{c2} corresponds to the onset of superconductivity, H^* is typically associated with onset of dissipation due to melting of the vortex lattice and the transition into the vortex liquid state [4, 5]. It appears that H_3S, displays a relatively narrow vortex liquid region, highlighted in cyan, particularly for the more homogeneous sample under 155 GPa, in contrast to unconventional high-T_c superconductors, which show a broad vortex liquid region and a convex $H^*(T)$ phase boundary [5], despite much higher transition temperatures in H_3S. The temperature range for the vortex liquid phase in the less homogeneous sample under 160 GPa is $\sim$ 25 K wide, but remains relatively unchanged at all fields.

REFERENCES

[1] Drozdov, A. P. *et al.* Superconductivity at 250 K in lanthanum hydride under high pressures. Preprint at https://arxiv.org/abs/1812.01561 (2018).

[2] Capitani, F. *et al.* Spectroscopic evidence of a new energy scale for superconductivity in H_3S. *Nature Physics* **13**, 859–863 (2017).

[3] Tajima, S. *et al.* Experimental study of electron–phonon interaction in MgB_2. *Physica C: Superconductivity* **388-389**, 103–104 (2003).

[4] Ramshaw, B. J. *et al.* Vortex lattice melting and H_{c2} in underdoped $YBa_2Cu_3O_y$. *Phys. Rev. B* **86**, 174501 (2012).

[5] Grissonnanche, G. *et al.* Direct measurement of the upper critical field in cuprate superconductors. *Nature Communications* **5**, 3280 (2014).
